# Effectiveness of the Beyond Good Intentions Program on Improving Dietary Quality Among People With Type 2 Diabetes Mellitus: A Randomized Controlled Trial

**DOI:** 10.3389/fnut.2021.583125

**Published:** 2021-03-05

**Authors:** Laura A. van der Velde, Jessica C. Kiefte-de Jong, Guy E. Rutten, Rimke C. Vos

**Affiliations:** ^1^Department of Public Health and Primary Care/LUMC-Campus The Hague, Leiden University Medical Center, The Hague, Netherlands; ^2^Department of General Practice, Julius Centre for Health Sciences and Primary Care, University Medical Centre Utrecht, Utrecht University, Utrecht, Netherlands

**Keywords:** healthy diet, self-management, diabetes mellitus type 2, randomized controlled trial, effect modifier, goals, patient education as topic

## Abstract

**Background and Aims:** An appropriate diet is an essential component of the management of Type 2 Diabetes Mellitus (T2DM). However, for many people with T2DM, self-management is difficult. Therefore, the Beyond Good Intentions (BGI) education program was developed based on self-regulation and proactive coping theories to enhance people's capabilities for self-management. The aim of this study was to determine the effectiveness of the BGI program on improving dietary quality among a preselected group of people with T2DM after two-and-a-half years follow-up.

**Methods:** In this randomized controlled trial, 108 people with T2DM were randomized (1:1) to the intervention (*n* = 56) (BGI-program) or control group (*n* = 52) (care as usual). Linear regression analyses were used to determine the effect of the BGI program on change in dietary quality between baseline and two-and-a-half years follow-up. In addition, potential effect modification by having a nutritional goal at baseline was evaluated. Multiple imputation (*n* = 15 imputations) was performed to account for potential bias due to missing data.

**Results:** According to intention-to-treat analysis, participants in the intervention group showed greater improvements in dietary quality score than participants in the control group (β = 0.71; 95%CI: 0.09; 1.33) after follow-up. Having a nutritional goal at baseline had a moderating effect on the effectiveness of the BGI program on dietary quality (*p*-interaction = 0.01), and stratified results showed that the favorable effect of the intervention on dietary quality was stronger for participants without a nutritional goal at baseline (no nutritional goal: β = 1.46; 95%CI: 0.65; 2.27 vs. nutritional goal: β = −0.24; 95%CI: −1.17; 0.69).

**Conclusions:** The BGI program was significantly effective in improving dietary quality among preselected people with T2DM compared to care as usual. This effect was stronger among participants without a nutritional goal at baseline. A possible explanation for this finding is that persons with a nutritional goal at baseline already started improving their dietary intake before the start of the BGI program. Future studies are needed to elucidate the moderating role of goalsetting on the effectiveness of the BGI program.

## Introduction

Type 2 Diabetes Mellitus (T2DM) and its complications largely contribute to the global disease burden, making T2DM one of the leading causes of global deaths (1). Complications of T2DM comprise an increased risk of neuropathy, retinopathy, and nephropathy (microvascular), and cerebrovascular-, ischemic heart-, and peripheral vascular diseases (macrovascular) (2). The main drivers in the global T2DM epidemic are lifestyle related factors, including obesity, a sedentary lifestyle, and an unfavorable diet (1). This highlights the importance of managing T2DM and prevent or delay complications, which can be achieved through good cardiometabolic control (3).

Good cardiometabolic control in T2DM can be accomplished by pharmacological treatment and/or lifestyle modification, with improving physical activity levels and dietary quality as main components (3). Improving a person's dietary quality can optimize glucose levels. High-quality diets (i.e., adequate consumption of whole grains, fruits, nuts, fibers, vegetables, and legumes, moderate alcohol consumption, and low consumption of saturated fats, oils, salts, meat products, and sugar), contribute to T2DM prevention and management (4, 5). An important element in achieving good cardiometabolic control is the self-management of people with T2DM (6). However, for many people with T2DM it is difficult to incorporate self-management into their daily lives. Therefore, diabetes self-management education programs, defined as a continuous process intended to facilitate knowledge, skills, and abilities that are needed for diabetes self-management (7), are recognized as essential elements in T2DM care.

Thoolen et al. (8) developed the self-management education program “Beyond Good Intentions” (BGI), which specifically targets initiation and maintenance of self-management. The BGI program was developed using a comprehensive theoretical framework based on theories of self-regulation and proactive coping (8). The BGI intervention was previously found to be effective in reducing cardiovascular risk after 12 months in newly detected people with T2DM, regardless of medical treatment (9). Further research into the long-term effectiveness of the BGI program in a preselected group of people with T2DM (with diagnosed T2DM for up to 5 years) showed no evidence for significant improvement in BMI, cardiovascular risk factors, quality of life, or diabetes self-management behavior after two-and-a-half years follow-up in the ELDES study (10). However, the (long-term) effectiveness of the BGI program on improving dietary quality has not yet been evaluated. Previous studies indicate that self-management programs can improve dietary quality in the short term, however, few studies have studied changes in dietary quality on the longer term (11). Therefore, this study aimed to evaluate the effect of the BGI program in the ELDES study on diet quality among a preselected group of people with T2DM after two-and-a-half years follow-up.

## Methods

### Study Design

The study had a parallel randomized controlled trial (RCT) design with a two-and-a-half years follow-up time, and was conducted according to the CONSORT guidelines for experimental designs (12). The study was approved by the Medical Ethical Committee of the University Medical Center Utrecht and was registered at the Dutch Trial Register (Netherlands Trial Register NTR5530/NL5405).

### Participant Recruitment, Selection, and Randomization

In the Netherlands, there are ~115 care groups that offer disease management programs for people with a chronic disease, including people with T2DM. The care groups are responsible for the primary care and for the prevention of complications and symptoms in people with T2DM (13). Of all the people with T2DM, 85% are treated by general practitioners (GPs) in practices close to their homes (14). Three care groups with 43 general practices (either single or group-handed practices) and 89 GPs agreed to participate. In the period between 2014 and 2016, people with T2DM were recruited from care groups in the Dutch city Eindhoven. The participating GPs determined the eligibility of people with T2DM based on the electronic medical records. Individuals were eligible to participate if they were (1) aged between ≥18 and ≤75 years, and (2) diagnosed with T2DM between 3 months and 5 years. An invitation letter and response card were sent to all eligible people. If applicable, they were further informed about the BGI program through a telephone conversation with a member of the research team. When the potential participant was sufficiently informed and fully understood the information, written informed consent was obtained where after preselection was started.

The preselection was conducted to select those people with T2DM who might benefit from the BGI program. Selection was based on individual's self-management capabilities, and absence of severe anxiety and/or depression (as this should be addressed first), using the validated Self-Management Screening (SeMaS) questionnaire (15). The patient's GP was informed about the results of the SeMaS. Selection procedures are described in more detail elsewhere (16).

Eligible individuals (*n* = 1,590) were enrolled in the study if they consented to participate and replied to the SeMaS questionnaire (*n* = 119). Patients were excluded based on the results of the SeMaS questionnaire or had an insufficient cognitive performance, or inadequate knowledge of the Dutch language (*n* = 11), resulting in a study population of *n* = 108. During the two-and-a-half years follow up, one participant died, one moved outside the study area, 21 participants did not respond to the second questionnaire, and 12 discontinued the BGI intervention (Supplementary Figure 1).

The 108 preselected people with T2DM were randomized (1:1) based on computer-generated random numbers, with sealed, opaque, sequentially numbered allocation envelopes. The coordinating research center (Julius Center, UMC Utrecht) allocated all preselected individuals to the intervention group (BGI program) (*n* = 56) or the control group (care as usual) (*n* = 52), based on a random number corresponding to one of the groups. Participants who were attending the BGI program could not be blinded for the treatment allocation since they knew that they were attending an extra program besides care as usual. Similarly, nurses who were trained to give the BGI program were not blinded. However, the researcher who performed the outcome measurements was blinded.

### Control: Care as Usual

Participants in the control group received usual diabetes care according to the guidelines of the Dutch College of General Practitioners (12). The GP remains ultimately responsible for the care of people with T2DM. Participants are fully informed about T2DM, various treatment options, and their individual treatment plan. GPs did not offer other diabetes self-management education programs to the participants during the study.

### Intervention: Beyond Good Intentions Program

The intervention group received not only care as usual, but also followed the BGI program. The main objective of the BGI program is to help people with T2DM achieve optimal self-management by making them more knowledgeable, proactive, and confident about their disease management. The BGI program lasts 12 weeks and consists of several components. First, a 30-min individual introductory session was held between the participant and a trained nurse, wherein participant's knowledge, experiences, and attitudes regarding T2DM management were discussed and participants were asked to set personal goal(s) for improving their diabetes risk profile before the start of the next session. The individual session was followed by four bi-weekly group sessions of two-and-a-half hours, wherein topics relevant to all people with T2DM were discussed and participants were asked to formulate, implement, and evaluate pro-active self-management goals. Topics that were discussed included physical activity, medication adherence, self-management and a healthy diet. Dietary intake recommendations were based on diabetes-specific dietary guidelines currently used in practice, i.e., limit saturated fat intake, increase unsaturated fat intake, and increase dietary fiber intake (especially form fruits and vegetables) (13, 17). Two weeks after the last group session participants filled out an evaluation form, which was discussed in an individual evaluation session. Additionally, 1 year after the first individual session, a group booster session was scheduled, wherein the progress in goal attainment over the past months was discussed and new personal goals for the future were set. The BGI program is described in detail by Vos et al. (16), and a program overview is summarized in Table 1.

**Table 1:** Beyond Good Intentions program overview.

**Program component**	**Description**
Individual session	•One session
	•Duration: 30 min
	•Participants: participant and trained nurse
	•Discussed: participant's knowledge, experiences, and attitudes regarding T2DM management
	•To do: set personal goals to improve diabetes risk profile before start group session
Group sessions	•Four sessions, biweekly
	•Duration: 2.5 h each
	•Discussed: topics relevant to all people with T2DM (including physical activity, healthy diet, medication adherence, and self-management)
	•To do: formulate, implement, and evaluate pro-active self-management goals
Individual evaluation session	•Two weeks after last group session participants filled out evaluation form, which was discussed in an individual evaluation session
Boosting session	•When: 1 year after first individual session
	•Discussed: progress in goal attainment over the past months
	•To do: develop new personal goals for the future

### Nurse Training

The BGI program was guided by registered nurses (*n* = 3), specialized in care for people with T2DM. Before the start of the BGI program, the nurses had two 3-h training sessions conducted by a psychologist who designed the original program of BGI, following the train-the-trainer principle. The nurses encouraged the participants in the BGI program to support each other and to gain more self-confidence in collecting information about T2DM themselves.

### Dietary Intake and Diet Quality Score

#### Dietary Intake Assessment

Dietary intake was assessed based on questions derived from two food-based dietary questionnaires; the Kristal food habits questionnaire (FHQ) (18) and the Dutch Fat Consumption Questionnaire (FCQ) (19). The FHQ consisted of 20 questions covering topics regarding participants' approaches to reduce fat intake. The questions were based on a four-point Likert scale, with answer options ranging from “never” to “always,” including a “not applicable” option. The FCQ consisted of 39 questions covering seven groups of food products (dairy, bread and spreads, meat, and cheese for warm dishes, dish-gravy, snacks, fruit, vegetables, and sugar-containing beverages). Participants were asked the frequency and sometimes also the quantity of the foods consumed (e.g., “How many days a week do you eat vegetables”). Questions about the frequency of consumption were based on seven or eight scales with answer options ranging from “never or less than once a month” to “7 times or more each week,” Questions about the number of servings a day were based on 4, 6, 8, or 10 answer options, ranging from “not applicable” to “9 times or more a day.”

#### Construction and Scoring of the Dietary Quality Score

The dietary quality score (DQS) used in the current study was based on an existing DQS developed by Nettleton et al. (20), specifically focused on people with T2DM and based on the intake of nine food groups: whole grains, vegetables, fruits, nuts, and fish (favorable food groups), and red and processed meats, snacks, sugar sweetened beverages, and sweets (unfavorable food groups). In the current study, dietary intake data of the FHQ and FCQ were used to develop an adapted version of the DQS. Because the dietary intake data were not completely comparable to the data used by Nettleton et al., two food groups were slightly modified: the “whole grains” food group was changed to “grains,” and the “fish” food group was changed to “fish and poultry as a replacement of red meat.” These food groups and the questions on which they were based are presented in Supplementary Table 1.

In the DQS developed by Nettleton et al., food group scores were divided into population-specific quartiles. Individuals were assigned points based on their quartile rank, where higher scores reflected better DQSs. Favorable food groups were assigned ascending scores (0–3 points); individuals in the highest intake quartile received 3 points. Unfavorable food groups were assigned descending scores (3–0 points); individuals in the highest quartile received 0 points.

In the adapted DQS, food group scores were divided into population-specific quartiles or medians. Food groups divided into median intakes were scored 1 or 2 points, food groups divided into quartile intakes were scored 0.5; 1; 1.5; or 2 points, with higher scores reflecting a better dietary quality (Supplementary Table 1). A minimal clinically important difference has not been quantified for the DQS. Favorable food groups were assigned ascending scores and unfavorable food groups were assigned descending scores. Most food groups were based on a single dietary intake item, however, the “red and processed meat” and “sweets” food groups were composed of multiple dietary intake items. For these food groups, each dietary intake item was separately distributed in population-specific quartile or median intakes and scored based on these distributions, where after the scores of these items were summed and divided by the number of items per food group. The total DQS was calculated as the sum of the food group scores, resulting in a total DQS ranging from a theoretical minimum of 6.5 to a theoretical maximum of 18 points on a continuous scale, where higher scores reflected a better dietary quality.

### Sociodemographic and Lifestyle Variables

Sociodemographic and lifestyle information was assessed at baseline (T0) and after two-and-a-half years follow-up (T1). Age, sex, paid employment status (yes/no), marital status (married/ divorced/widow or widower/ never married), currently having a nutritional goal (yes/no), dietary intake, and physical activity (hours per day light to heavy activity) were assessed using self-reported questionnaires at T0 and T1. Educational level (low/ intermediate/ high education) was retrieved from the self-reported SeMaS questionnaire. In addition, glycated hemoglobin (HbA1c), systolic blood pressure (SBP), lipid profile [low-density lipoprotein (LDL)-cholesterol, high-density lipoprotein (HDL)-cholesterol, total cholesterol, and triglycerides], smoking status (current/former/never smoker), and height and weight of the participants were retrieved from the electronic medical records of the GP. Body Mass Index (BMI: kg/m^2^) was calculated from the height and weight stated in the GP electronic medical records.

### Potential Effect Modification by Nutritional Goals

Having a nutritional goal was identified as a potential effect modifier, based on clinical plausibility and previous literature (21). For example, a systematic review showed that participants who set goals prior to a diabetes self-management intervention improved their glycemic control after the intervention (22). A possible explanation for this finding might be that people who set goals have a higher motivation to improve their glycemic control (23). Furthermore, participants that set a nutritional goal may be more focused on their diet and have more nutrition knowledge. Persons with more nutrition knowledge are more likely to consume healthier diets (24). Therefore, it is plausible a different effect of the BGI program on DQS could be expected for participants with a nutritional goal compared to those without a nutritional goal.

### Statistical Analysis

Baseline participant characteristics were described using descriptive statistics. Continuous variables were described as means and standard deviations (SD) when normally distributed, or medians and interquartile range (IQR) when non-normally distributed. Categorical variables were reported as frequencies and percentages.

For the DQS construction, the intake of food groups was divided into population-specific quartiles or medians with the use of the “Visual binning” option, which is a tool to assist the researcher in transforming continuous variables into categorical variables. The difference in DQS between baseline (T0) and follow-up (T1) measurement was calculated as the DQS at T1 minus the DQS at T0. In a similar manner, differences in food group scores between baseline and follow-up were calculated as the food group score at T1 minus the food group score at T0. Linear regression analyses were performed according to the intention-to-treat principle to examine the effect of the BGI intervention on DQS and food group scores between T0 and T1, presented as the change in DQS for the intervention group compared to the control group and adjusted for baseline values of dietary quality. Effect modification by nutritional goals was examined by adding a product term “intervention^*^nutritional goal” to the regression model. If this interaction was significant, analyses were repeated and results on the effect of the BGI intervention on DQS was shown separately for those with and without a nutritional goal.

As a sensitivity analysis, a non-response analysis was conducted to examine potential differences between baseline characteristics of participants who had missing data on the dietary intake questions (non-respondents) and participants who had complete data on the dietary intake questions (respondents). Missing data were imputed (*n* = 15 imputations) using the multiple imputation procedure in SPSS with the predictive mean matching method (25), with the exception of the nutritional goal and dietary quality questions. Because results were similar in original and imputed data (Table 2), results of the main analyses were described after the multiple imputation procedure. All statistical analyses were performed using IBM SPSS Statistics for Windows version 25.0 (IBM Corp., Armonk, N.Y., USA). A two-sided *p*-value of <0.05 was considered statistically significant.

**Table 2:** Participant characteristics in original and imputed data, split by intervention and control group.

	**Data after multiple imputation (*****n =*** **108)**		**Original data (*****n =*** **108)**			
**Characteristics**	**Intervention (*n =* 56)**	**Control (*n =* 52)**	**Intervention**		**Control**	
			***n***		***n***	
Age (years)	62.89 ± 8.30	61.71 ± 7.44	56	62.89 ± 8.30	52	61.71 ± 7.44
Sex, male	27 (48.2)	33 (63.5)	56	27 (48.2)	52	33 (63.5)
Educational level			56		52	
Low	15 (26.8)	16 (30.8)		15 (26.8)		16 (30,8)
Intermediate	20 (35.7)	17 (32.7)		20 (35.7)		17 (32.7)
High	19 (33.9)	16 (30.8)		19 (33.9)		16 (30.8)
Other	2 (3.6)	3 (5.8)		2 (3.6)		3 (5.8)
Marital status, married	36 (64.3)	40 (76.9)	55	36 (64.3)	51	40 (78.4)
Paid employment	16 (28.6)	22 (42.3)	55	16 (29.1)	51	21 (41.2)
Nutritional goal at baseline^a^, yes	26 (46.4)	25 (48.1)	47	26 (46.4)	42	25 (48.1)
Smoking status			56		52	
Current	4 (7.1)	6 (11.5)		4 (7.1)		6.11.5)
Former	31 (55.4)	22 (42.3)		31 (55.4)		22 (42.3)
Never	21 (37.5)	24 (46.2)		21 (37.5)		24 (46.2)
BMI, kg/m^2^	29.52 ± 4.85	30.07 ± 4.55	55	29.58 ± 4.87	52	30.07 ± 4.55
HbA1c, mmol/mol	49.14 ± 7.36	49.79 ± 8.69	54	49.13 ± 7.47	52	49.79 ± 8.69
SBP, mmHg	131.45 ± 13.43	133.35 ± 14.47	55	131.87 ± 13.17	52	133.35 ± 14.47
Lipid profile, mmol/l						
LDL cholesterol	2.60 ± 0.84	2.37 ± 0.83	55	2.60 ± 0.85	52	2.37 ± 0.83
HDL cholesterol	1.27 ± 0.28	1.18 ± 0.37	49	1.26 ± 0.27	47	1.19 ± 0.36
Total cholesterol	4.60 ± 0.89	4.14 ± 0.92	49	4.58 ± 0.89	47	4.09 ± 0.88
Triglycerides	1.60 (1.13; 2.08)	1.60 (1.13; 2.08)	55	1.60 (1.20; 2.10)	52	1.60 (1.20; 2.08)
Physical activity, hours per week	10.88 (5.44; 23.25)	12.00 (3.56; 24.00)	53	10.50 (5.00; 22.38)	47	13.50 (3.75; 24.00)
Dietary quality score^a^	12.83 ± 1.71	12.91 ± 1.97	48	12.83 ± 1.71	49	12.91 ± 1.97

*Data are n (%), mean ± Standard deviation (SD), or median [interquartile range (IQR)], data of imputation number 15 are presented for the data after multiple imputation*.

a *Nutritional goal at baseline (yes/no) was not imputed: Intervention (n = 47), Control (n = 42); Diet quality score was not imputed: Intervention (n = 48), Control (n = 49)*.

## Results

### Subject Characteristics

Most baseline participant characteristics were similar between the intervention and control group (Table 2). Participants had a mean age around 62 years and were overall well-controlled. The DQS at baseline was comparable between the intervention and control group [intervention: 12.8 (± 1.7); control: 12.9 (± 2.0)]. By chance, the control group had a larger percentage of participants that were male (intervention: 48.2%; control: 63.5%), married (intervention: 64.3%, control: 76.9%), and currently employed (intervention: 28.6%; control: 42.3%; Table 2).

### Effect of the BGI Program on Dietary Quality

Change in DQS after the intervention according to intention-to-treat analyses is shown in Table 3. Forty-eight participants had missing data for at least one of the dietary intake questions that determine the total DQS. Therefore, data on change in DQS were available for 60 participants. Total DQS significantly improved for the intervention group compared to the control group after the intervention period (β = 0.71, 95%CI = 0.080; 1.33, *p* = 0.028), indicating that participants in the intervention group had a 0.71 points higher DQS change after the intervention compared to the control group. Of the individual components, only fruit intake significantly improved after the intervention period (β = 0.23, 95%CI = 0.02; 0.44, *p* = 0.033). The components vegetables and nuts also improved after the intervention period, although not significantly, while for the other components minimal changes were observed (Table 3).

**Table 3:** Change in dietary quality and dietary quality components after the intervention for the intervention group relative to the control group (intention to treat analysis).

	**Change in dietary quality for the intervention group compared to the control group**	**95% Confidence Interval**
	**β^a^**	
**Total dietary quality score**		
	0.71	0.080 to 1.33^*^
**Individual dietary quality components**		
Grains	−0.057	−0.28 to 0.17
Fish	−0.017	−0.20 to 0.17
Snacks	−0.073	−0.29 to 1.17
Nuts	0.094	−0.12 to 0.31
Fruits	0.23	0.015 to 0.44^*^
Vegetables	0.18	−0.016 to 0.37
Meat	0.074	−0.039 to 0.19
Sweets	−0.065	−0.29 to 0.16
Sugar containing beverages	0.068	−0.032 to 0.17

a *Coefficients are adjusted for baseline values of dietary quality/ individual component scores and indicate the change in DQS for the intervention group compared to the control group*.

* *P < 0.05*.

### Sensitivity and Subgroup Analyses

Most baseline characteristics were comparable between respondents (*n* = 60) and non-respondents (*n* = 48) to the dietary intake questions (Supplementary Table 2). Non-respondents to the dietary intake questions generally had a slightly higher BMI compared to respondents in both the intervention and control group. Compared to respondents, non-respondents in the control group had a slightly higher mean SBP. Further, they more often had a nutritional goal, and more often were male (Supplementary Table 2).

**Figure 1:**
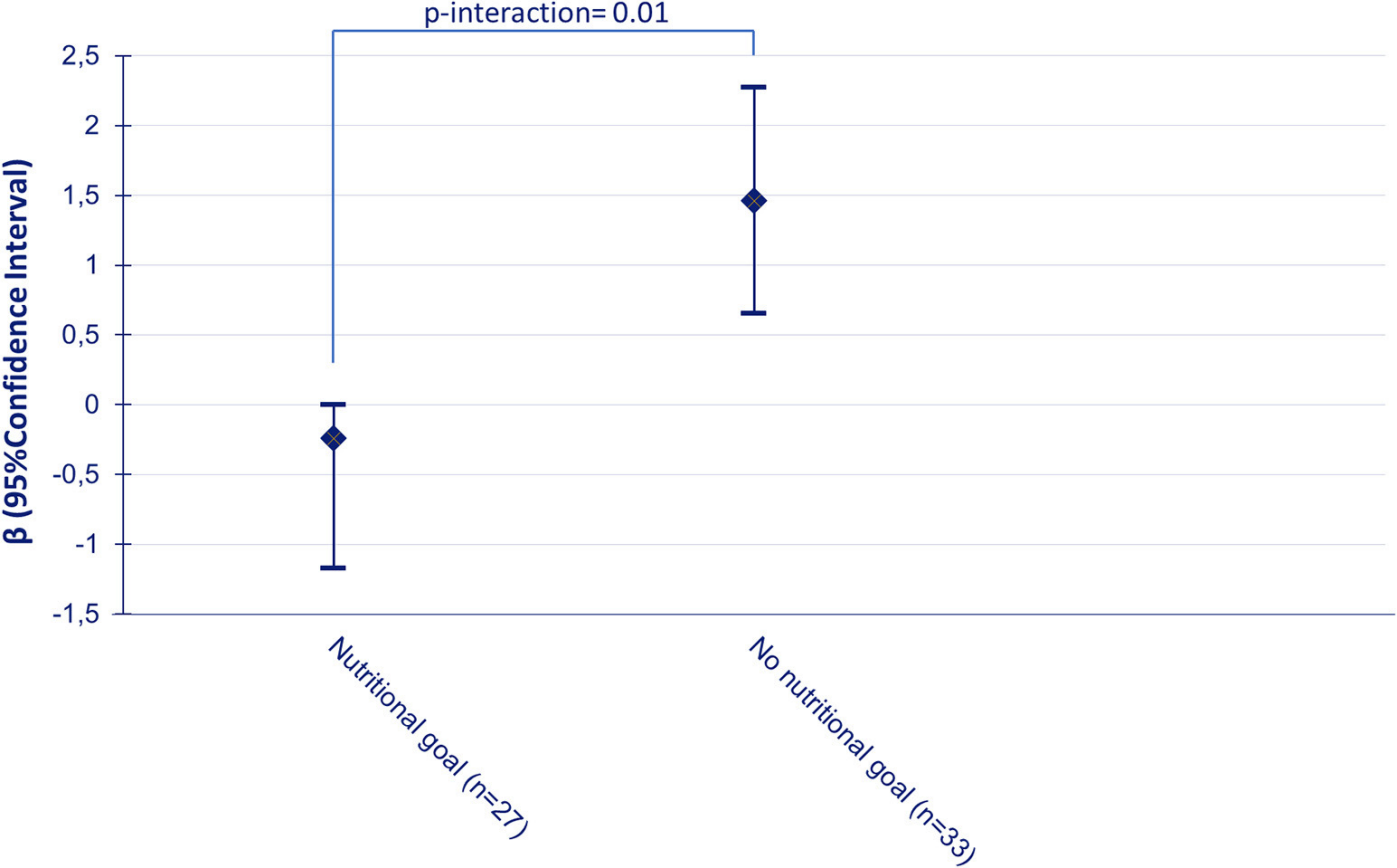
Change in dietary quality score for the intervention group relative to the control group between baseline and follow-up according to nutritional goalsetting at baseline, adjusted for baseline values of dietary quality.

Having a nutritional goal at baseline was found to have a moderating effect on the effect of the intervention on DQS (*p*-interactio*n* = 0.01), and stratified results showed that the favorable effect of the intervention on DQS was stronger for participants without a nutritional goal at baseline (β = 1.46; 95%CI: 0.65; 2.27 vs. −0.24; 95%CI: −1.17; 0.69) (Figure 1).

## Discussion

People with T2DM who followed the BGI program showed greater improvements in DQS than those only receiving care-as-usual after two-and-a-half years follow-up. This result was mainly driven by an increased fruit consumption. The favorable effect of the BGI program on DQS was stronger for participants without a nutritional goal at baseline.

A systematic review by Norris et al. (11) shows that self-management education in people with T2DM generally has a positive influence on dietary intake. For example, the study by Glasgow et al. (26) including 206 people with diabetes aged 62 years on average, showed a positive effect of a self-management education program on reducing fat and energy intake after 3 months compared to care as usual. However, most reviewed studies had a short follow-up (≤1 year) and did not take into account total dietary quality (11). Elaborating on these previous studies, our study is among the first to demonstrate longer-term effectiveness of a self-management education program on improving dietary quality. We found an improvement in DQS of ~0.7 units after participation in the BGI program compared to the control group (care as usual). To illustrate the clinical relevance of these findings, the observed difference in DQS should be compared with the assigned points for each food group: individuals were assigned 0,5; 1; 1,5; or 2 points for each food group with higher scores indicating better adherence to the dietary guidelines. Our finding of a 0.7 difference in the DQS between the intervention and control group is comparable to a 0.5 to 1 point increase for one food group. For example, an individual who consumed snacks more than one time a week (0.5 points) decreased their snack consumption to once every 2 or 3 weeks (1 point) or once a month (1.5 points).

An unexpected finding is the stronger positive effect of the program on DQS in participants without a specific nutritional goal. According to the goal-setting theory, defining goals facilitates behavior change by guiding individuals' devotion and effort which promotes persistence to overcome barriers, and increases self-efficacy for self-management in general (27). A systematic review by Fredrix et al. (22) showed that participants who set goals prior to a diabetes self-management education program improved their glycemic control after the intervention, suggesting that goal-setting could be a beneficial strategy for improving glycemic control. Collaborative goal-setting when receiving medical care for diabetes can improve glycemic control through greater perceived self-management competence and an increased level of trust in the physician (28). Furthermore, a study including 54 overweight people with T2DM with diagnosed T2DM ≥ 1 year, and at least one additional risk factor for CVD, showed that nutritional goal setting within a self-management intervention improved dietary quality, assessed using the Healthy Eating Index 2010 (29). Based on these results one would expect that the intervention would be more effective among those with a nutritional goal at baseline, whereas we found the opposite.

A possible explanation might be that some people with T2DM already started improving their dietary intake after diagnosis. This hypothesis is in line with the results of a previous study among 144 newly diagnosed people with T2DM in the Netherlands, which showed that these people had an unfavorable fat intake at time of diagnosis compared to the general population, but adapted to a more favorable fat intake shortly after diagnosis and maintained this more favorable intake for 4 years (30). It might be that participants who were already more focused on their diet were also more likely to set a nutritional goal, even though they already improved their diet upon diagnosis, which would leave less room for improvement in DQS following the intervention. Because the current study lacked data on the exact time of diagnosis, future studies are warranted to further explore this hypothesis.

To the best of our knowledge, this study is the first to assess longer-term effectiveness of a self-management education program on improving dietary quality among preselected people with T2DM. The results of this study need to be interpreted in the context of its strengths and limitations.

Our study is strengthened by its design. Randomized controlled trials (RCTs) are viewed as the golden standard for studying cause-effect relations (31). However, even in RCTs, missing data can lead to biased results and thereby threaten validity of inferences (32). Therefore, we applied multiple imputation (25), a recommended method for dealing with missing data in RCTs (32). However, for nutritional goal and dietary intake data it cannot be assumed that data are missing at random (MAR) which is an important assumption to justify imputation. Therefore, for these variables only observed data was used (32).

Some methodological considerations regarding the DQS construction in our study need to be discussed. Deviating from the score of Nettleton et al., we did not distinguish whole grain from refined grain products, because this data was not available. Because whole-grain products have health benefits that are lacking in refined grain products, and because only whole-grain products are protective of T2DM (33), it would have been preferred to include only whole-grain products in our DQS (34). Furthermore, the fish food group deviated from the original score as poultry was included. Although this is not completely comparable to the original DQS, both fish and poultry consumption are considered favorable, and red meat unfavorable components in most DQSs (34). In line with current national dietary guidelines, we based our DQS on the intake of food groups instead of individual nutrients, because persons consume a combination of several foods instead of individual nutrients, and because this takes into account interactions of nutrients within food products (35). Although this approach does not take into account heterogeneity in for example nutritional values within food groups, using food groups is the preferred approach for DQS construction in the context of public health promotion (35). Further, we chose to use population-specific percentile (median and quartile) cut-offs, which do not necessarily reflect healthy intake levels, but enabled a good discriminatory power for each food group (34, 35). Although it may have been useful for the interpretation of the results of the current study, no minimal clinically important difference in DQS could be quantified because there is insufficient scientific evidence to support such a quantification. However, to illustrate the clinical relevance of our results demonstrating a difference in improvement in DQS of 0.7 units after participation in the BGI program, the observed difference in DQS should be compared with the aforementioned assigned points for each food group [i.e., a 0.7 difference in the DQS between the intervention and control group is comparable to a 0.5 to 1 point increase for one food group, such as decreasing the consumption of snacks from more than one time a week (0.5 points) to once every 2 or 3 weeks (1 point) or once a month (1.5 points)].

Several variables included in the current study were retrieved from GP electronic medical records, which limits biases generally associated with self-reporting such as social desirability bias (36). Our primary outcome measure (dietary quality) was, however, assessed based on self-reported dietary intake data. These data were collected using food frequency questionnaires (FFQs), which is the most commonly used data collection tool for determining dietary quality together with 24-h recall and dietary records (37). Although widely accepted, FFQs have some limitations, such as their limited amount of included food items, and sensitivity to measurement error (38). As stated above, self-reporting of dietary intake is prone to social desirability bias (36). Additionally, participants in the intervention group may have reported more favorable intakes because they knew that they were receiving nutritional education.

Lastly, the inclusion rate of the current study was low (7.5%), which may have been attributable to the recruitment by invitation letter rather than recruitment by personal invitation, and the time and effort required from participants in the intervention group (10). In addition, although most characteristics were comparable between responders and non-responders, some differences were observed. Therefore, the results of this study may not be generalizable to the total population of people with T2DM.

In conclusion, the BGI program was effective in improving DQS among preselected people with T2DM after two-and-a-half years follow-up and could therefore contribute to good cardiometabolic control in people with T2DM. The favorable effect of the BGI program on DQS was stronger for participants without a nutritional goal at baseline, possibly because participants that had set a nutritional goal before the start of the study were already more focused on their diet and had already started improving their diet, leaving less room for improvement in DQS following the intervention. Future studies are needed to elucidate the moderating role of setting a nutritional goal on the effectiveness of the self-management education program on DQS, and to evaluate whether the BGI program is also effective in improving DQS among different subgroups of people with T2DM, such as those with comorbidity or poorly controlled T2DM.

## Data Availability Statement

The raw data supporting the conclusions of this article will be made available by the authors upon request, without undue reservation.

## Ethics Statement

The studies involving human participants were reviewed and approved by the Medical Ethical Committee of the University Medical Center Utrecht. The patients/participants provided their written informed consent to participate in this study.

## Author Contributions

GR was the principle investigator of the trial. RV was the trial coordinator. JK, RV, and LV were involved in drafting the statistical analysis plan. LV drafted the manuscript, in close collaboration with JK and RV. All authors read, edited, and approved the final manuscript.

## Conflict of Interest

The authors declare that the research was conducted in the absence of any commercial or financial relationships that could be construed as a potential conflict of interest.
