# Supplementary material for: Effectiveness of the Beyond Good Intentions Program on Improving Dietary Quality Among People With Type 2 Diabetes Mellitus: A Randomized Controlled Trial
Source: Front Nutr. 2021 Mar 5;8:583125. doi: 10.3389/fnut.2021.583125 (PMC7973042; doi:10.3389/fnut.2021.583125)
Supplement: Supplementary file 1 [file Table_1.pdf]

Supplementary Table 1. Food groups included in the dietary quality score, their corresponding question(s), and allocated points based on population-specific quartile or median intakes

	Question (Q)	Points per quartile or median (lowest to highest intake)			
		**1 (lowest intake)**	**2**	**3**	**4 (highest intake)**
			**1 (lowest intake)**		**2 (highest intake)**
Favorable food groups					
Grains	Q1:	How many servings of bread, crackers, or toast do you eat a day			
	Quartile points:	0.5	1	1.5	2
Vegetables	Q2:	How many times a week do you consume vegetables			
	Median points:		1		2
Fruit	Q3:	How many times a week do you consume fruits			
	Median points:		1		2
Nuts	Q4:	How often do you eat peanuts or nuts			
	Quartile points:	0.5	1	1.5	2
Fish and poultry*	Q5:	How often do you consume fish or poultry instead of red meat			
	Median points:		1		2
Unfavorable food groups					
Red/ processed meat	Q6:	How often do you eat cooked sausage with your hot meal			
	Median points:		2		1
	Q7:	How often do you eat half-and-half minced meat with your hot meal			
	Quartile points:	2	1.5	1	0.5
	Q8 :	How often do you eat strips of bacon with your hot meal			
	Median points:		2		1
	Q9:	How many slices of bread do you eat with meat products/ cold cuts			
	Median points:		2		1
Snacks	Q10:	How often do you eat snacks, like fries, croquettes			
	Quartile points:	2	1.5	1	0.5
Sugar-sweetened beverages	Q11:	How many glasses of soda, lemonade or sport drinks do you drink a day			
	Median points:		2		1
Sweets	Q12 t/m Q17:	How often do you eat: cake, pie, large cookies/ ice-cream/ candy bars, like Nuts, Mars, Snickers/ chocolate/ biscuits or raisin-biscuits/ other cookies			
	Quartile points:	2	1.5	1	0.5

* Fish and poultry as a replacement of red/processed meat
