# Supplementary material for: Effectiveness of the Beyond Good Intentions Program on Improving Dietary Quality Among People With Type 2 Diabetes Mellitus: A Randomized Controlled Trial
Source: Front Nutr. 2021 Mar 5;8:583125. doi: 10.3389/fnut.2021.583125 (PMC7973042; doi:10.3389/fnut.2021.583125)
Supplement: Supplementary file 2 [file Table_2.pdf]

Supplementary Table 2. Baseline characteristics of respondents and non-respondents to the dietary intake questions, split by intervention and control group

Characteristics	**Respondents (n=60)**		**Non-respondents (n=48)**	
	Intervention (n=28)	Control (n=32)	Intervention (n=28)	Control (n=20)
Age (years)	62.50 ±8.47	61.06 ±7.44	63.29± 8.26	62.75± 7.511
Sex, male	14 (50.0)	17 (53.1)	13 (46.4)	16 (80.0)
Educational level				
Low	11 (39.3)	9 (28.1)	4 (14.3)	7 (35.0)
Intermediate	9 (32.1)	12 (37.5)	11 (39.3)	5 (25.0)
High	7 (25.0)	10 (31.3)	12 (42.9)	6 (30.0)
Other	1 (3.6)	1 (3.1)	1 (3.6)	2 (10.0)
Marital status. married	21 (75.0)	26 (81.3)	15 (53.6)	14 (70.0)
Paid employment. employed	5 (17.9)	13 (40.6)	11 (39.3)	9 (45.0)
Nutritional goal[a]. yes	12 (50.0)	30 (48.1)	14 (50.0)	12 (60.0)
Smoking status				
Current	1 (3.6)	2 (6.3)	3 (10.7)	4 (20.0)
Former	14 (50.0)	16 (50.0)	17 (60.7)	6 (30.0)
Never	13 (46.4)	14 (43.8)	8 (28.6)	10 (50.0)
BMI. kg/m2	28.17± 4.00	29.57± 4.31	30.87± 5.30	30.86± 4.93
HbA1c. mmol/mol	50.07± 7.30	50.53± 10.10	48.21± 7.45	48.60± 5.82
SBP. mmHg	131.86± 14.96	130.00± 14.04	131.04± 11.97	138.70± 13.83
Lipid profile. mmol/l				
LDL cholesterol	2.67±0.85	2.28± 0.77	2.53± 0.84	2.51± 0.93
HDL cholesterol	1.27 ±0.29	1.18± 0.33	1.26± 0.28	1.20± 0.43
Total cholesterol	4.75± 0.90	4.10± 0.90	4.45± 0.87	4.22± 0.98
Triglycerides	1.60 (1.05; 2.10)	1.65 (1.23; 2.10)	1.65 (1.13; 2.00)	1.55 (1.13; 1.98)
Physical activity. hours per week	9.13 (3.19; 22.81)	8.25 (3.56; 24.56)	13.13 (6.00; 24.94)	17.25 (3.75; 23.63)
Diet quality score (T=0)[b]	12.74 ±1.74	13.16 ±1.91	12.98 ± 1.70	12.46± 2.04

Data are n (%), mean ± SD, or median (interquartile range). Data of Imputation number 15 are presented.

[a] Nutritional goal not imputed: Respondents: Intervention (n=24), Control (n=27); Non-respondents: Intervention (n=23), Control (n=15)

[b] Diet quality score not imputed: Respondents: Intervention (n=28), Control (n=32); Non-respondents: Intervention (n=20), Control (n=17)
