# Supplementary figures and images for: Effectiveness of the Beyond Good Intentions Program on Improving Dietary Quality Among People With Type 2 Diabetes Mellitus: A Randomized Controlled Trial
Source: Front Nutr. 2021 Mar 5;8:583125. doi: 10.3389/fnut.2021.583125 (PMC7973042; doi:10.3389/fnut.2021.583125)

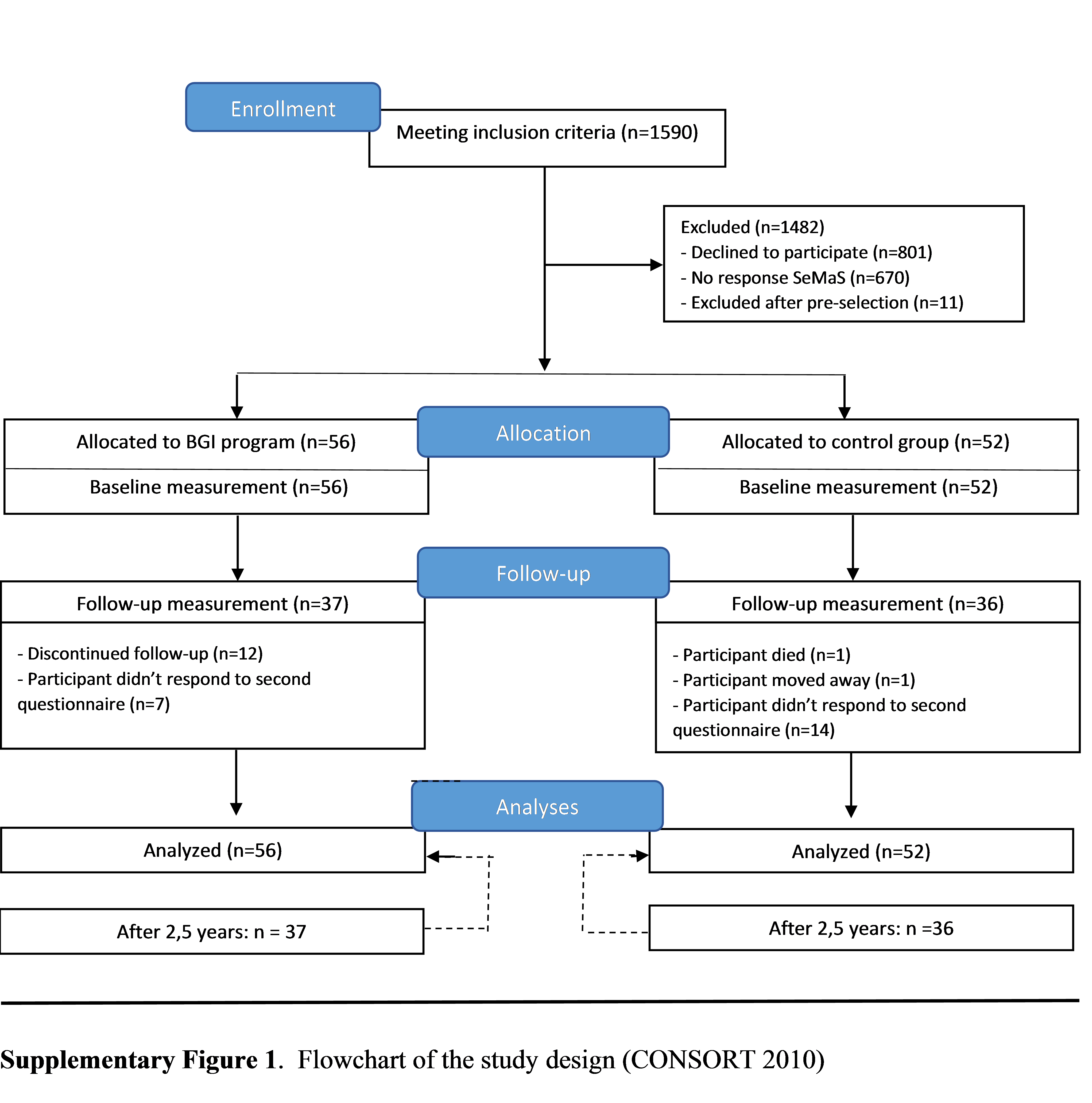

Supplement: Supplementary file 3 [file Image_1.tif]
